# Psychiatric and legal considerations for ketamine treatment within prison settings

**DOI:** 10.3389/fpsyt.2024.1316108

**Published:** 2024-04-18

**Authors:** Michael Bayrhammer-Savel, Martin Ortner, Marie-Claire Van Hout, Arkadiusz Komorowski

**Affiliations:** ^1^Department of Psychiatry and Psychotherapy, Medical University of Vienna, Vienna, Austria; ^2^Central Public Prosecutor’s Office for Combating Economic Crimes and Corruption, Vienna, Austria; ^3^Faculty of Health, Liverpool John Moores University, Liverpool, United Kingdom

**Keywords:** psychiatry, prison, human rights, mental health, ketamine, suicidality, treatment resistant depression

## Abstract

The fundamental right to equivalence of health care in prison settings encompasses the provision of medication to address mental health conditions. Considering the increased risk for self-harm among individuals dealing with depression, the limited effectiveness of conservative antidepressants is a major challenge in psychiatry. The high prevalence of suicidal tendencies within prison populations underscores the imperative for state-of-the-art pharmacological treatment to uphold adequate health care standards. Notably, the denial of access to effective medication could be deemed a violation of human rights of people living in prison according to international treaties, domestic law, and United Nations normative standards of detention. This article presents the authors’ perspective on the accessibility of ketamine treatment in prison settings, discussing psychiatric and legal considerations as well as current challenges in this context. Implementing novel psychopharmacological interventions may alleviate the distress experienced by individuals struggling with depressive symptoms and suicidality. At the same time, unprecedented treatment alternatives bring along potential issues, including limited understanding of long-term effects and the risk of abuse. Given the scarce data-availability, a pressing need exists for further research on the benefits and risks of ketamine treatment within prison populations.

## 1 Introduction

The global prison population is estimated to exceed 11.5 million, and people living in prison (PLP) commonly face challenges that impact their health, social reintegration, and life expectancy (1, 2). While elevated rates of communicable diseases result in higher mortality rates (3), a large part of the prison population is also affected by a psychiatric disorder (4, 5). Besides complex mental health conditions like attention-deficit/hyperactivity disorder (ADHD), posttraumatic stress disorder (PTSD), and substance use disorder (SUD), major depression appears to be particularly relevant (6, 7). Studies conducted in prisons show a prevalence of 35 - 38% for depressive disorders and a three to ninefold increase in suicide risk (8, 9), partly due to inadequate access to specialist treatment and environmental health stressors (10, 11). PLP represent a high-risk group, with reported rates of 18% and 31% for a lifetime history of self-harm and suicide attempts, respectively (12).

PLP are, in addition to the obvious restriction of their fundamental right to freedom, also exposed to potential violations of their right to physical and mental health as well as, in case of inadequate treatment of suicidality, their right to life. In order to address the health needs of those affected, penal authorities must strive for a level of care that matches the applicable standards in the general population (13, 14). The “principle of equivalent medical care” can be derived from a variety of human rights treaties, United Nations (UN) normative standards of detention, domestic legislation and policy, as well as international court decisions. Accordingly, novel treatment options may present an opportunity to provide adequate health care to a population typically underserved by the penal system. Considering the existing literature, we present a viewpoint on the potential access of prison populations to ketamine for the treatment of depression and suicidality.

### 1.1 The role of ketamine in psychiatry

In Art. 1, the *Convention on the Rights of Persons with Disabilities* (CRPD) delineates disability as a physical or mental impairment, which significantly limits one or more essential life activities. Within the scope of chronic psychiatric disorders, this may include self-care, concentration, cognitive functions, social interactions, communication, and vocational engagement. Comparable to other mental health conditions, clinically significant depressive episodes should be treated through an interdisciplinary approach, including pharmacological treatment. Commonly utilized antidepressants are selective serotonin reuptake inhibitors, serotonin-norepinephrine reuptake inhibitors, norepinephrine-dopamine reuptake inhibitors, and other classes of medications such as tricyclic antidepressants (15). However, in up to 30% of the cases, individuals do not respond adequately to the initial treatment (16). While clinicians occasionally prescribe an augmentation therapy with antipsychotics or lithium to enhance efficacy in treatment-resistant depression (TRD), polypharmacy increases the risk of adverse events and lithium exhibits a potential risk of hypothyroidism or kidney damage (17).

In line with the legal framework’s definition of a disability, individuals with TRD encounter noticeable functional impairment due to an insufficient response to antidepressant treatment. For such cases, the N-methyl-D-aspartate (NMDA) receptor antagonist ketamine stands as a viable alternative, given its differing pharmacodynamic profile compared to conventional antidepressants. It is currently hypothesized that ketamine binding triggers diverse molecular cascades which induce synaptic plasticity (18). Significant antidepressant efficacy and persistent effects during treatment has been confirmed repeatedly, creating strong evidence for its use (19–21). Ketamine has also emerged as a promising treatment option for individuals with suicidal ideation due to its rapid-acting effect (22, 23). Given the genetic covariance between major depression and suicide attempts, ketamine may constitute a personalized treatment alternative for persons whose genetic characteristics suggest a higher risk for these conditions (24). Within the context of psychiatric care, recent studies have further indicated a therapeutic potential of ketamine for individuals dealing with trauma-related conditions such as PTSD (25). However, to our knowledge, there are no studies assessing the effects of ketamine treatment in prison settings. Although alternative interventions designed to reduce depressive symptoms and suicidality of PLP, such as group-based treatment, psychoeducational and peer support programs, as well as individual psychotherapy, are widely implemented, current pharmacological and non-pharmacological treatment options are still insufficiently evaluated (26).

### 1.2 The principle of equivalence of care

The integration of scientific evidence into the penal system is vital, as correctional institutions are inherently bound by a moral and legal obligation to establish robust governance and effectively tackle health care issues. In its explanatory factsheet regarding Art. 25 of the *Universal Declaration of Human Rights* (UDHR), the UN specifies that “States have an obligation to prohibit and eliminate discrimination on all grounds and ensure equality to all in relation to access to health care and the underlying determinants of health” (27). The UN General Assembly Resolution *United Nations Standard Minimum Rules for the Treatment of Prisoners (The Nelson Mandela Rules)* states in Rule 24.1 that “prisoners should enjoy the same standards of health care that are available in the community, and should have access to necessary health-care services free of charge without discrimination on the grounds of their legal status” (28). Likewise, the World Health Organization recognized the status of PLP as a disadvantaged group and agreed on the fact that it is a public health issue for states to ensure same standards of health care inside prisons and outside (29, 30). Even further reaching, the CRPD mandates that public entities need to enact reasonable modifications in their policies, practices, or procedures whenever these adjustments are essential to prevent discrimination rooted in disability (31).

Similar to the UN standards, the Council of Europe (CoE) recommendations *European Prison Rules* state that “all necessary medical, surgical and psychiatric services including those available in the community shall be provided to the prisoner” (32). In all countries that have acceded to the CoE, the strongest legislative sword is the *European Convention on Human Rights* (ECHR): Any person that claims to be violated in a right stipulated by the ECHR is permitted to apply to the European Court of Human Rights (ECtHR) for a chamber ruling, and the Committee of Ministers of the CoE is obligated to enforce the chamber’s decision. The ECtHR derives from Art. 2 ECHR (Protection of Life) the duty of member states to protect prisoners from suicide or self-harm (33). Furthermore, in Art. 3 ECHR (Torture and Inhumane Treatment), it is stipulated that “no one shall be subjected to torture or to inhuman or degrading treatment or punishment”. One of the most relevant rulings of the ECtHR regarding the treatment of PLP is the 2013 grand chamber judgment *Murray v. The Netherlands* (34). While the applicant was sentenced to life imprisonment for murder, a release from prison was held in prospect de jure, as long as the individual’s risk of reoffending was mitigated. However, since the applicant was not provided appropriate psychiatric treatment, Murray claimed he had no chance of being released de facto. The ECtHR followed his claim and ruled that The Netherlands had violated Art. 3 ECHR. The court pointed out that “states are under an obligation to provide detainees suffering from health problems – including mental health problems – with appropriate medical care”. Another widely known example of where the ECtHR chamber recognized a violation of Art. 3 ECHR by a member state is the 2016 judgment *Wenner v. Germany* (35). In this case, the ECtHR reiterated the principle of equivalence of care, under which prisoners were entitled to medical treatment in conditions comparable to those enjoyed by patients in the outside community.

Already in 1976, the US Supreme Court (SCOTUS) issued the landmark decision *Estelle v. Gamble* (36), finding a violation of the Eighth Amendment (Cruel and Unusual Punishment) due to “deliberate indifference by prison personnel to a prisoner’s serious illness or injury” (37). This ruling also set the legal foundation for the right to receive opioid agonist treatment (OAT) within prison settings in the US. Among others, the judicial rulings *Pesce v. Coppinger* (38), *Smith v. Aroostook County* (39), and *Kortlever v. Whatcom County* (40) confirmed, that OAT must be offered, as long as it is available to the general public as well. Considering the legal implications of failing to do so, future debate needs to address the implementation of novel pharmacological treatments in prisons.

### 1.3 Methodological considerations

Introducing novel treatments to PLP encompass methodological and ethical challenges, particularly in low- and middle-income countries (7, 41). It is crucial to acknowledge that PLP inherently encounter vulnerable circumstances, often compounded by power differentials (42). Voluntariness and individual autonomy can be compromised by institutional pressure, even if subliminal, exemplified by unethical historical research in prison settings (43, 44). Consequently, studies conducted in prisons are essential to explore the specific situation and needs of PLP (45).

Balancing the risk of harm with potential benefits, PLP should be granted access to participation in clinical and other research under the principle of equivalence of care (46, 47). Despite system barriers and, in some cases, a lower health literacy of prison populations (48), the capacity to provide informed consent as a research participant is not universally precluded (49). In addition to being carefully scrutinized by an ethical review committee, studies involving prison populations should also adhere to general research guidelines. International principles governing the conduct of health research include the Declaration of Helsinki by the World Medical Association (50) as well as the International Ethical Guidelines for Health-related Research Involving Humans (51) and Epidemiological Studies (52) by the Council for International Organizations of Medical Sciences. Comparable to the general population, treatment recommendations for PLP should be based on scientific evidence, such as double-blind randomized controlled trials alongside comprehensive meta-analytical findings. However, the fact that PLP are insufficiently represented in health research complicates data-driven decision-making (3, 45). Studies in prisons are often hampered by discontinuity of care and tight financial constraints (53, 54), which limits longitudinal research on the effectiveness of clinical interventions.

## 2 Discussion

While penal authorities are legally compelled to provide treatment when it is available to the general public as well, PLP commonly face disparities in access to mental health care (4, 6, 13). Although restrictive measures have been linked to the exacerbation of self-inflicted harm as well as psychological distress, penal authorities often deploy solitary confinement in response to acute crises (55, 56). Individuals experiencing suicidal ideation may therefore benefit from novel treatment options. There has been only gradual progress in the provision of state-of-the-art treatment within prison settings. E.g., medication prescription patterns for a given pathology still differ between prison and community settings (57), and ketamine treatment has come to the authors’ attention as an example of this discrepancy. However, as the clinician’s perspective often overlooks necessary adjustments for organizing health care within the prison environment, reasonable concerns about the role of ketamine for PLP necessitate further considerations.

Balancing the goal of offering adequate health care with the need to prevent disruptive behaviors can create conflicts of interest for medical professionals. Given inconsistent findings in prisons, adherence to guidelines and implementation of periodic monitoring due to the risk of adverse effects is considered crucial (58). Although benzodiazepines should not be used as a long-term treatment due to their side effects including cognitive impairment and dependence, prison populations frequently receive this medication with the intention of bridging the onset of action of antidepressants and antipsychotics (57). Regarding schizophrenia, the implications of equivalent medical care extend to the administration of clozapine due to its unparalleled effectiveness (59). The use of clozapine among individuals with treatment resistant schizophrenia shows an overall response rate of approximately 40% in the general community (60). While empirical data is scarce, more frequent prescriptions of clozapine result in significant reductions of disciplinary measures and segregation in prisons (61). In contrast, medical treatment of ADHD in forensic settings is still controversially discussed (62), even though ADHD is estimated to affect at least 20% of PLP (63). While prior randomized controlled trials have provided encouraging results (64), a recent study has shown no short-term effects of methylphenidate in male PLP (65). Similarly, the use of OAT in prisons remains a contentious issue in many countries, although research suggests that it is associated with a reduction in overall mortality and drug-related poisonings following release (66). Using OAT within the domain of opioid use disorder is not only broad consensus in the scientific community, but also a legal imperative (67, 68). Turning to the domain of depression, odds-ratios between 1.37 and 2.13 are estimated for pharmacological treatments in the general population (69). While reported effect sizes for clinical interventions in prisons range between 0.17 and 1.41, the effectiveness of antidepressants is still insufficiently studied in PLP (70). In the context of non-pharmacological treatment, cognitive behavioral treatment and mindfulness-based interventions are commonly recommended in prison settings (4, 71). However, these treatment programs have not demonstrated superior effects compared to other psychological therapies. It should be taken into consideration that PLP commonly display heightened levels of psychopathology alongside increased instances of comorbidity, complicating the evaluation of clinical interventions. Further reasons for the slow advances in adoption of novel treatment options relate to the planning efforts of research studies, the administration of psychotropic medication in prison environments, and financial constraints (8, 9, 45, 54).

While there are no studies investigating ketamine specifically, awareness regarding the potential abuse and adverse effects holds significant importance (72, 73). Clinicians need to address the high-risk behaviors linked to prison populations such as drug exchange and injection, leading to an elevated risk of symptom exaggeration in order to gain access to the medication. Compared to the general population, PLP tend to have a higher rate of SUD, with a prevalence ranging between 10 and 50% (6, 74). This shows the utmost importance of careful consideration of pharmacological treatment alternatives for TRD and suicidality. Notably, substance abuse within the prison environment is known for multiple pharmaceuticals such as benzodiazepines and gabapentinoids that are routinely prescribed during acute crises (57). Thus, it is prudent to limit access and require ketamine administration be closely supervised and monitored in a controlled setting by qualified medical personnel. With regard to ketamine, a nasal spray for application of esketamine, the S(+) enantiomer of ketamine, has recently been approved for therapeutic use in several countries, minimizing the risk of substance misuse and side effects such as dissociation (75, 76). Although there is strong evidence supporting the positive benefit related to suicide ideation for intravenous racemic ketamine, studies investigating intranasal esketamine did not yet show the same favorable profile (77, 78). Given the current lack of empirical evidence, it therefore seems too early to draw conclusions regarding potential effects of esketamine in prison settings.

Regarding future directions, examining how ketamine treatment aligns with established international legal precedents seems reasonable. In the past, the ECtHR has repeatedly dealt with complaints from PLP who brought forward issues related to inadequate diagnoses and treatments (34, 35). The ECtHR generally holds that states have an obligation to ascertain the health condition of prisoners through examinations and to promptly provide them with appropriate treatments when needed (79). I.e., “the Court considers that, for the purposes of Art. 3 of the Convention, it is not sufficient for the detainee to be examined and diagnosed. To safeguard the health of the prisoner, it is essential that therapy corresponding to the established diagnosis and appropriate medical supervision be carried out” (80). Denying access to ketamine treatment while exclusively offering conventional antidepressants may further constitute a form of discrimination based on disability as defined by the CRPD, specifically targeting individuals with TRD. To administer ketamine within prisons wherever indicated is not only in line with current medical standards but, in our opinion, also coincides with the principle of equivalence.

In conclusion, the persistent disregard for research on prison health carries substantial consequences for mortality rates, disease prevention, and the fundamental right to health. It is imperative to consider not only the legal dimensions, as expounded upon here, but also the medical implications (59, 67). The scarcity of studies exploring disorder-specific pharmacological interventions within the prison environment can be attributed to methodological complexities, such as monitoring follow-up and selecting appropriate outcome measures (46, 53). In view of the fact that penal authorities need to offer even better health services than those available to the general population to reach comparable health outcomes (14, 66), we advocate the investigation of ketamine treatment for PLP dealing with depression and suicidal ideation under controlled conditions. The denial of access to effective evidenced-based medication can potentially lead to a violation of the prohibition of inhuman or degrading treatment and, in the case of suicide, to a violation of the most fundamental basic right, the right to life.

## Data availability statement

The original contributions presented in the study are included in the article/supplementary material. Further inquiries can be directed to the corresponding author.

## Author contributions

MB-S: Conceptualization, Writing – original draft, Writing – review & editing. MO: Writing – review & editing. M-CH: Writing – review & editing. AK: Conceptualization, Supervision, Writing – original draft, Writing – review & editing.
